# Gender Specific Mutation Incidence and Survival Associations in Clear Cell Renal Cell Carcinoma (CCRCC)

**DOI:** 10.1371/journal.pone.0140257

**Published:** 2015-10-20

**Authors:** Christopher J. Ricketts, W. Marston Linehan

**Affiliations:** Urologic Oncology Branch, Center for Cancer Research, National Cancer Institute, National Institutes of Health, Bethesda, Maryland, United States of America; Florida State University, UNITED STATES

## Abstract

Renal cell carcinoma (RCC) is diagnosed in >200,000 individuals worldwide each year, accounting for ~2% of all cancers, but the spread of this disease amongst genders is distinctly uneven. In the U.S. the male:female incidence ratio is approximately 2:1. A potential hypothesis is mutation spectra may differ between tumors dependent upon the gender of the patient, such as mutations of X chromosome encoded genes being more prevalent in male-derived tumors. Combined analysis of three recent large-scale clear cell renal cell carcinoma (CCRCC) mutation sequencing projects identified a significantly increased mutation frequency of *PBRM1* and the X chromosome encoded *KDM5C* in tumors from male patients and *BAP1* in tumors from female patients. Mutation of *BAP1* had previously been significantly associated with poorer overall survival; however, when stratified by gender, mutation of *BAP1* only significantly affected overall survival in female patients. Mutation of chromatin remodeling genes alters gene regulation, but the overall effect of these alterations may also be modified by the presence of other gender specific factors. Thus, the combination of gender and mutation of a specific gene, such as *BAP1*, may have implications not only for prognosis but also for understanding the role of chromatin remodeling gene mutations in kidney cancer progression.

## Introduction

Renal cell carcinoma (RCC) is diagnosed in >200,000 individuals in the world each year, accounting for ~2% of all cancers, but the incidence of this disease amongst genders is distinctly uneven. In the U.S. between the years of 2001–2010 the age-adjusted incidence rate per 100,000 cancers of the kidney and renal pelvis for males increased from 17.5–20.6 and for females increased from 8.6 to 10.0; however, the ratio of incidence between males and females has remained at approximately 2:1 (ranging from 1.78:1–2.07:1) [1]. This gender bias is not unique to RCC, for instance, a similar gender bias is observed in bladder cancer in the U.S. where between the years of 2001–2010 the male to female ratio has remained at approximately 4:1 (ranging from 3.87:1–4.21:1) [1]. There are currently no known causes for this bias. The recent reports of large scale sequencing and integrated analyses of clear cell RCC (CCRCC), the most common type of RCC (representing ~75% of all cases), provides a large amount of mutation data for CCRCC samples, allowing for the investigation of potential hypotheses [2–4].

One such hypothesis is the potential for gender specific differences in the rate of X chromosome mutation between male and female patients. In males, each cell contains a single X chromosome whilst the complimentary Y chromosome contains similar versions of only a fraction of the genes present on the X chromosome. Thus, a single inactivating mutation of an X chromosome gene in a male patient results in complete loss of gene activity. In females, each cell contains two X chromosomes, one of which is largely inactivated at a very early stage of embryogenesis. Thus, for the majority of genes, a single inactivating mutation would have to occur in the gene on the activated X chromosome to result in loss of gene activity, the only exception being the small number of genes that escape X inactivation. However, this potential mechanism is dependent upon X chromosome gene mutation being observed in, and being relevant to, the carcinogenesis of CCRCC.

Recent studies of CCRCC have highlighted the importance of mutation in chromatin remodeling/modifier genes in CCRCC tumorigenesis, including components of the SWI/SNF complex such as *PBRM1*, *ARID1A* and *SMARCA4* and histone residue modifiers such as *SETD2*, *KDM5C* (*JARID1C*) and *KDM6A* (*UTX*). While several of these most commonly mutated chromatin remodeling/modifier genes are present on chromosome 3p, a chromosomal region that is lost in a high percentage of CCRCC tumors, three of these genes, *KDM5C* (*JARID1C*), *KDM6A* (*UTX*) and *STAG2*, are located on the X chromosome [2,5]. In this study the distribution of X chromosome and other genomic alterations was compared between tumors derived from male and female patients. Three recent reports of CCRCC were used to compile a dataset of 628 tumors for use in this analysis.

## Methods

### Ethics Statement

All patient data was acquired from three previously published studies and each study stated that written informed consents were obtained.

The TCGA KIRC cohort report by the TCGA stated in their methods that “Specimens were obtained from patients, with appropriate consent from institutional review boards”. This data is available in volume 499 of Nature on pages 43–49 [2]. TCGA data concerning genomic and clinical information is organized into two categories: one that is openly accessible to the public and one that has controlled access, available only to qualified researchers obligated to secure the data. The open access data set contains only information that is not individually unique and does not pose a risk of patient re-identification. All the data used within this manuscript was obtained from the open access data set and has passed the criteria for unrestricted publication with the following statement “No restrictions; all data available without limitations” listed at http://cancergenome.nih.gov/abouttcga/policies/publicationguidelines.

The Japanese cohort reported by Sato *et al* stated in their methods that “Paired tumor-normal DNA was isolated from 240 ccRCC specimens and subjected to comprehensive molecular analyses after written informed consent was obtained. This study was approved by the ethics committee of the Graduate School of Medicine at the University of Tokyo.” This data is available in volume 45 of Nature Genetics on pages 860–867 [3].

The Chinese cohort reported by Guo *et al* stated in their supplementary methods that “A signed written consent from each patient was obtained before the recruitment in the study according to the regulations of the institutional ethics review boards.” This data is available in volume 44 of Nature Genetics on pages 17–19 [4].

### Data Retrieval

All the TCGA KIRC cohort patient clinical and mutation data was accessed from the online open access Nature article entitled “Comprehensive molecular characterization of clear cell renal cell carcinoma” in volume 499, pages 43–49 by selecting the supplementary information [2]. The supplementary information contained in files entitled “Data_File_S2_clinical_dataset.xlsx” and “Data_File_S10_KIRC-BCM-BI-UCSC-gapfill-v1.11.txt” contains all the data used herein.

All the Sato *et al* cohort patient clinical and mutation data was accessed from the online Nature Genetics article entitled “Integrated molecular analysis of clear-cell renal cell carcinoma” in volume 45, pages 860–867 by selecting the supplementary information [3]. The supplementary information contained in files entitled “Supplementary table 1. Characteristics of the patients” and “SupplementaryTable 3. List of mutations in exome-sequencing” contains all the data used herein.

All the Guo *et al* cohort patient clinical and mutation data was accessed from the online Nature Genetics article entitled “Frequent mutations of genes encoding ubiquitin-mediated proteolysis pathway components in clear cell renal cell carcinoma” in volume 44, pages 17–19 by selecting the supplementary information [4]. The supplementary information contained in files entitled “Supplementary Text and Figures” and “Supplementary Table 5” contains all the data used herein.

All mutation data retrieved from these three source studies was collated and tabulated into S1 Table.

### Data Analysis

Fisher’s exact tests were used to compare the rates of mutation for each gene between the two genders with a p-value of <0.05 considered indicative of a positive trend and a p-value of <0.005 considered statistically significantly due to Bonferroni correction. Kaplan-Meier survival curves were calculated using the cBioPortal software (http://www.cbioportal.org/index.do) [6,7]. The cBioPortal software was used to assess the mutation status of genes within the TCGA bladder cohort, produce oncoprints, and assess the mutual exclusivity of mutations within the cohorts.

## Results

To evaluate whether gender bias was present, the mutation rate per gender was assessed for ten genes commonly mutated in CCRCC consisting of the 3 genes present on the X chromosome (*KDM5C*, *KDM6A* and *STAG2*), the 4 genes present within the commonly lost and mutated chromosome 3p region (*VHL*, *PBRM1*, *SETD2* and *BAP1*) and the *ARID1A* (chr.1p), *PTEN* (chr.10q) and *TP53* (chr.17p) genes. These genes were assessed by combining the data from three recent studies of CCRCC tumors consisting of cohorts representing patients from the U.S.A., Japan and China that all demonstrated a higher degree of male patients. The U.S.A. based cancer genome atlas (TCGA) clear cell kidney cancer (KIRC) report consisted of 424 sequenced samples with 277 males and 147 females (male/female ratio 1.88:1) [2]. The Japanese cohort reported by Sato *et al* consisted of 106 sequenced samples with 78 males and 28 females (male/female ratio 2.79:1) [3]. The Chinese cohort reported by Guo *et al* consisted of 98 sequenced samples with 59 males and 39 females (male/female ratio 1.51:1) [4]. This produced a total of 628 sequenced samples with 414 males and 214 females (male/female ratio 1.93:1) that were investigated for nine of the ten genes (excluding *STAG2*) and 530 sequenced samples (male/female ratio 2.03:1) that were investigated for *STAG2* due to an absence of data in the Guo *et al* publication.

**Fig 1:**
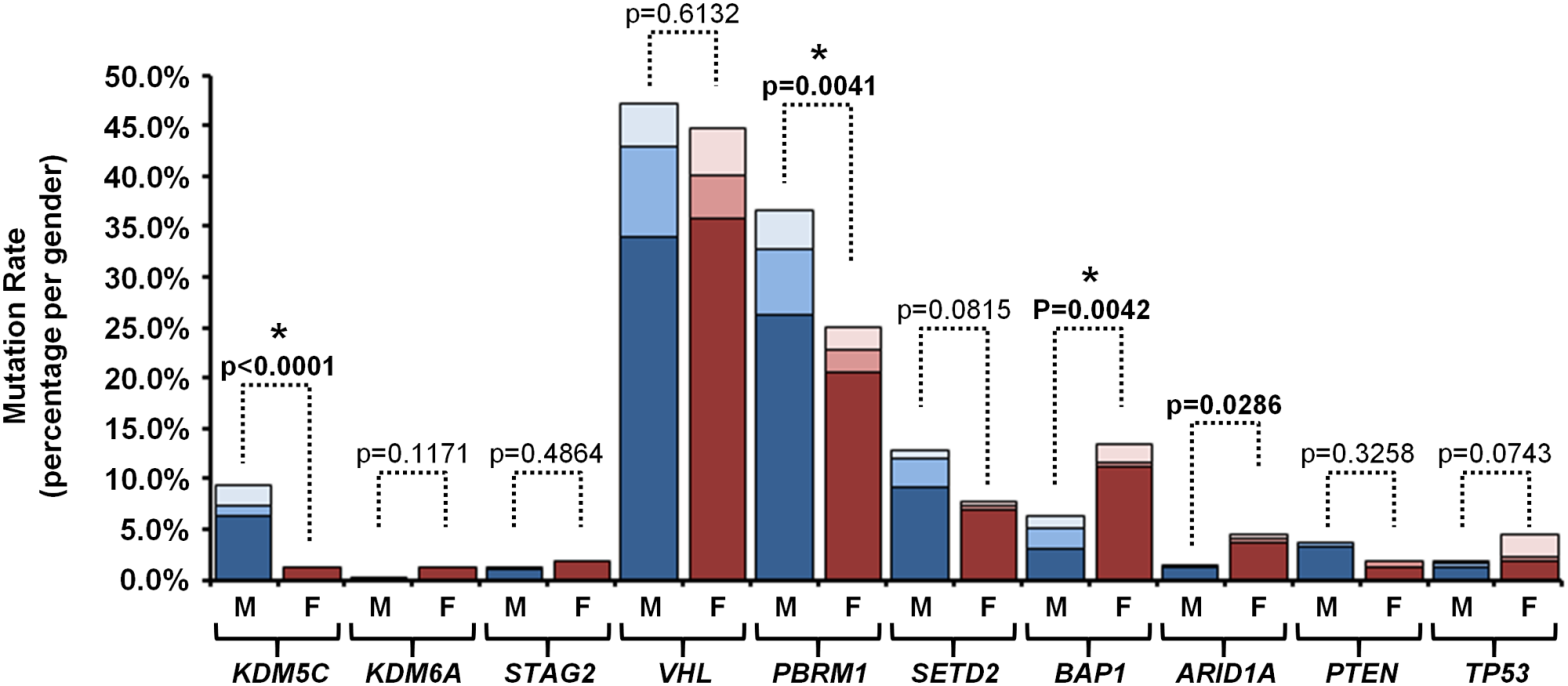
The Gender Specific Mutation Rate of 10 Commonly Mutated CCRCC Genes. The percentage of mutation for 10 genes commonly mutated in CCRCC genes were collated for 628 tumors from three large sequencing projects (*STAG2* data was only available from two projects) [2–4]. The difference in mutation frequency between genders was assessed by Fisher’s Exact tests. Bold text represents statistically significant differences before Bonferroni correction (p-value < 0.05) and * represents statistically significant differences after Bonferroni correction (p-value < 0.005). The expected percentage of mutations in male or female patients was calculated based on the overall mutation rate and the ratio of males to females. The blue bars represent the male patients with dark blue signifying the TCGA cohort, blue signifying the Sato *et al* cohort and light blue signifying the Guo *et al* cohort. The red bars represent the female patients with dark red signifying the TCGA cohort, red signifying the Sato *et al* cohort and light red signifying the Guo *et al* cohort.

The combined cohort demonstrated no difference in mutation frequency between male or female derived tumors for 6 out of ten genes, including two of the X chromosome genes, *KDM6A* and *STAG2* (Fig 1 and Table 1). Two genes, *BAP1* and *ARID1A*, demonstrated an increased mutation rate in female derived tumors, but only *BAP1* was considered statistically significant after Bonferroni correction (p = 0.0042) (Fig 1 and Table 1). Notably, this trend was statically significant in the TCGA KIRC cohort alone (p = 0.0001), but was not observed in either of the other two cohorts (Table 1), possibly a reflection of the differing genetic backgrounds present in these ethnically different cohorts. The combined *BAP1* mutation rate in CCRCCs was 8.8% in comparison to 6.3% in males and 13.6% females (Fig 1). Two genes, *KDM5C* and *PBRM1*, demonstrated a statistically significant increase in mutation rate in male derived tumors with p-values of p<0.0001 and p = 0.0041 respectively. The trend for *KDM5C* was observed in all three cohorts with no *KDM5C* mutations observed in any female derived tumors in two of the cohorts and the remaining TCGA KIRC cohort demonstrated only 3 out of 29 *KDM5C* mutations in female derived tumors and a statistically significant trend of increased mutation rate in male derived tumors (p = 0.0039) (Table 1). The combined *KDM5C* mutation rate in CCRCCs was 6.7% in comparison to 9.4% in males and 1.4% females (Fig 1). The trend for *PBRM1* was present in all three cohorts; however it was only the combination of these cohorts that demonstrated statistical significance (Table 1). The combined *PBRM1* mutation rate in CCRCCs was 32.8% in comparison to 36.7% in males and 25.2% females (Fig 1).

**Table 1:** Analysis of Mutation Frequency in 10 Genes Commonly Mutated in CCRCC.

		Chromosome X			Chromosome 3p				Chr. 1	Chr. 10	Chr. 17
Mutated Gene		*KDM5C*	*KDM6A*	*STAG2*	*VHL*	*PBRM1*	*SETD2*	*BAP1*	*ARID1A*	*PTEN*	*TP53*
**All Cohorts**	Total	42 (6.7%)	4 (0.6%)	9 (1.4%)	291 (46.3%)	206 (32.8%)	70 (11.1%)	55 (8.8%)	16 (2.5%)	19 (3.0%)	18 (2.9%)
	Male	39	1	5	195	152	53	26	6	15	8
	Female	3	3	4	96	54	17	29	10	4	10
	Fisher's Exact	**<0.0001**	0.1171	0.4864	0.6132	**0.0041**	0.0815	**0.0042**	**0.0286**	0.3258	0.0743
**TCGA KIRC**	Total	29 (6.8%)	4 (0.9%)	8 (1.9%)	218 (51.4%)	153 (36.1%)	53 (12.5%)	37 (8.7%)	13 (3.1%)	17 (4.0%)	9 (2.1%)
	Male	26	1	4	141	109	38	13	5	14	5
	Female	3	3	4	77	44	15	24	8	3	4
	Fisher's Exact	**0.0039**	0.1223	0.4566	0.8383	0.0567	0.3555	**0.0001**	0.0706	0.1929	0.5047
**Sato *et al***	Total	4 (3.8%)	0 (0.0%)	1 (0.9%)	46 (43.4%)	32 (30.2%)	13 (12.3%)	9 (8.5%)	2 (1.9%)	2 (1.9%)	3 (2.8%)
	Male	4	0	1	37	27	12	8	1	1	2
	Female	0	0	0	9	5	1	1	1	1	1
	Fisher's Exact	0.5712	1.0000	1.0000	0.1875	0.1489	0.1767	0.4397	0.4604	0.4604	1.0000
**Guo *et al***	Total	9 (9.2%)	0 (0.0%)	n/a	27 (27.6%)	21 (21.4%)	4 (4.1%)	9 (9.2%)	1 (1.0%)	0 (0.0%)	6 (6.1%)
	Male	9	0	n/a	17	16	3	5	0	0	1
	Female	0	0	n/a	10	5	1	4	1	0	5
	Fisher's Exact	**0.0104**	1.0000	n/a	0.8194	0.1312	1.0000	1.0000	0.3980	1.0000	**0.0354**

The greatest increase in mutation rate in male derived tumors was observed in the X chromosome encoded *KDM5C* gene, but the other two X chromosome genes, *KDM6A* and *STAG2*, demonstrated no gender bias and the *BAP1* and *PBRM1* genes that additionally demonstrated gender bias are both on chromosome 3. This suggests that the chromosomal position of the mutated gene may only be one factor in determining any mutation rate differences and could be gene specific. To further assess the potential for both gender bias in mutation rate and a correlation between mutation of X chromosome encoded genes and male derived tumors a similar analysis was performed on the TCGA bladder urothelial carcinoma (BLCA) cohort. The TCGA BLCA cohort consisted of 128 tumors derived from 96 male patients and 32 female patients (male:female ratio = 3:1) and eight genes were evaluated including the same three X chromosome encoded genes (*KDM5C*, *KDM6A* and *STAG2*), four additional chromatin remodeling/modifier genes (*ARID1A*, *SETD2*, *MLL2* and *MLL3*) and *TP53*. This demonstrated no increased mutation rate for X chromosome encoded genes in male derived tumors or statistically significant increases in mutation rate associated with either gender (S2 Table).

**Fig 2:**
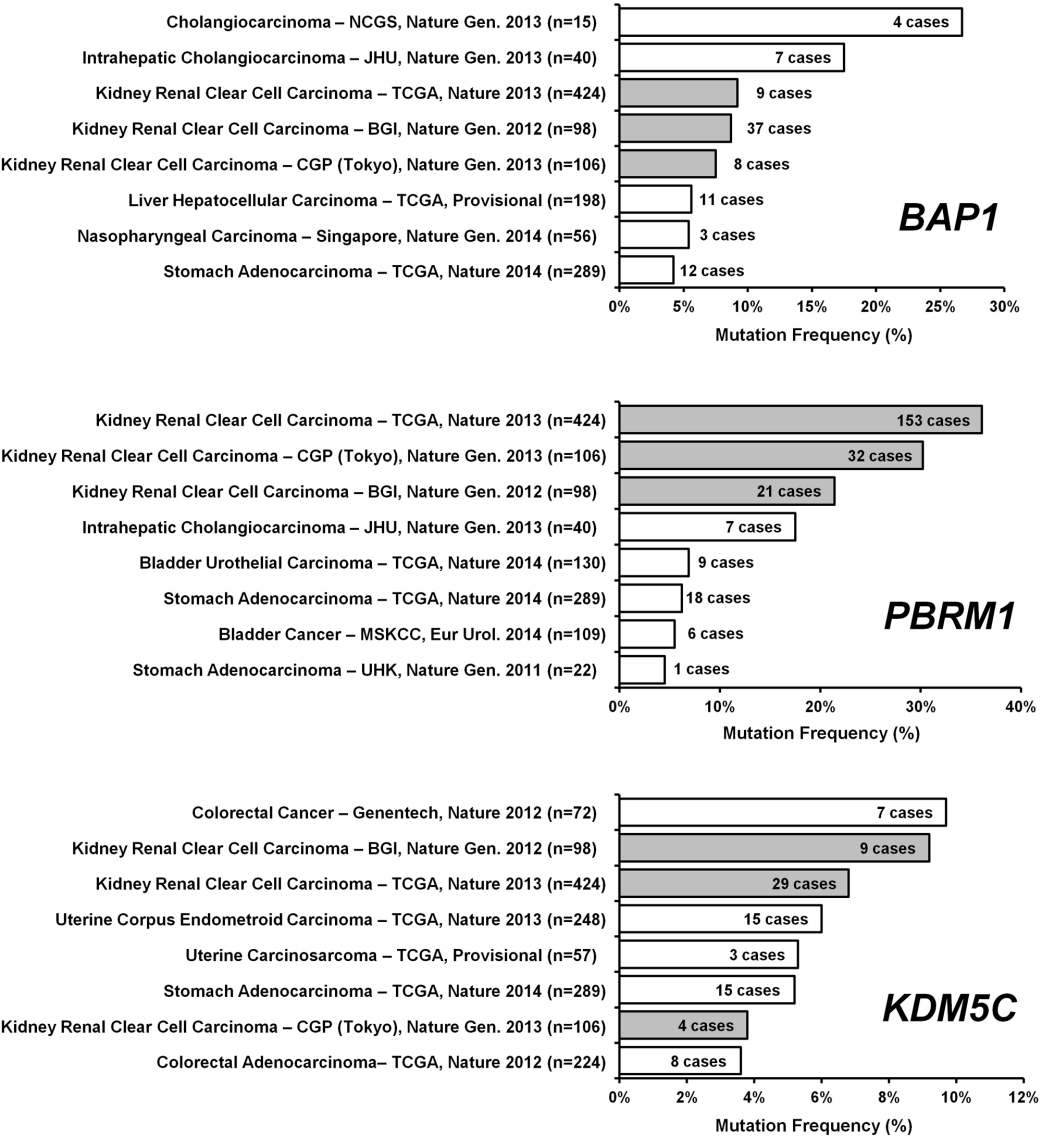
Comparative Mutation Frequencies of CCRCC-Associated Genes in Cancer. The relative mutation frequencies of *PBRM1*, *BAP1* and *KDM5C* in the three CCRCC cohorts were compared to the large number of cancer genome studies listed within the cBioPortal software (http://www.cbioportal.org/index.do) [6,7]. The three CCRCC cohorts were highlighted in grey with the TCGA cohort referred to as TCGA, Nature 2013, the Sato *et al* cohort referred to as CGP (Tokyo), Nature Gen. 2013 and the Guo *et al* cohort referred to as BGI, Nature Gen. 2013.

The three genes that demonstrated significant gender specific mutation rates in CCRCC; *KDM5C*, *PBRM1* and *BAP1*, also represent genes that are highly associated with this type of cancer. Comparison of the three cohorts with the large number of cancer sequencing projects (n = ~70) available on the cBio Portal [6,7] website were assessed using cBio Portal software and demonstrated that these three cohorts represent the three highest mutation frequencies of *PBRM1* mutation, the 3^rd^ to 5^th^ highest mutation frequencies of BAP1 mutation and the 2^nd^, 3^rd^ and 7^th^ highest mutation frequencies of *KDM5C* mutation (Fig 2). An assessment of mutual exclusivity between these ten selected genes within the TCGA KIRC cohort also demonstrated that *BAP1* and *PBRM1* were the only genes that demonstrated a statistically significant tendency towards mutual exclusivity (S1 Fig). This mutual exclusivity was more pronounced in the male patients where no tumors had mutation of both genes, in contrast, in the female patients 8 out of the 24 tumors with *BAP1* mutations also harbored *PBRM1* mutations (S1 Fig). This is suggestive of a similar resultant effect of losing either gene, especially in males, and in combination with a potential selection bias between the genders, that this resultant effect is more potent with *PBRM1* loss in males and *BAP1* loss in females.

**Fig 3:**
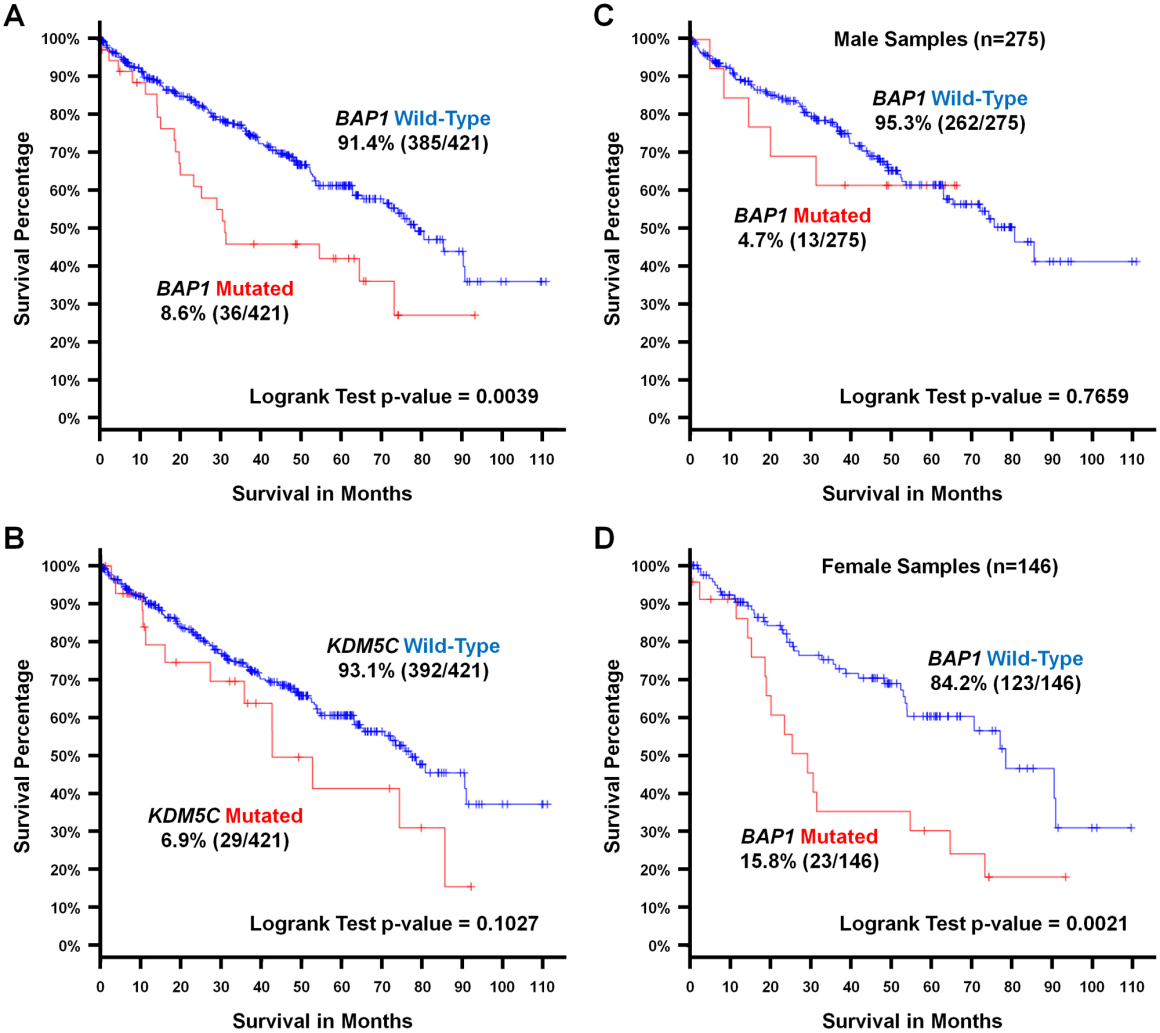
Mutation and Gender Specific Survival Analysis. (A and B) Kaplan-Meier survival analysis graphs were produced comparing samples with or within mutation of either *BAP1* or *KDM5C* for CCRCC TCGA cohort and (C and D) comparing the differences in survival with or without mutation of *BAP1* for each gender. Analysis was performed using the cBioPortal software (http://www.cbioportal.org/index.do) [6,7] and mutated samples were represented by red lines and wild-type samples by blue lines. Survival data was available for 421 samples within the CCRCC TCGA cohort consisting of 275 males and 146 females. p-values less than 0.01 were considered statistically significant.

In addition to their strong association with CCRCC, mutation of these chromatin remodeling/modifier genes has been associated with poorer patient survival and higher stage disease. The survival data was publically available for the TCGA KIRC cohort and mutation in these three genes was assessed in total and by gender using the cBio Portal software. Comparison of male and female patients demonstrated no difference in survival between the two genders (p = 0.516) (S2 Fig). Mutation of *BAP1* was associated with a significantly poorer survival in total (p = 0.0039) and in female patients (p = 0.0021), but not in male patients (p = 0.7659) (Fig 3). Whereas, mutation in either *PBRM1* or *KDM5C* did not associate with a statistically significant effect on patient survival in total or by gender (Fig 3 and S2 Fig).

## Discussion

The observation of gender bias in clear cell renal cell carcinoma is likely to be the result of multiple factors including genetic and environmental, as well as the differing basic physiology between males and females, such as differing levels of hormones. Increased mutation rate within X chromosome encoded genes in male patients presents as one potential genetic factor and this analysis suggests that this may be true for specific X chromosome genes, such as *KDM5C*, but was not universally observed for the X chromosome genes associated with CCRCC. The observed significance of the *KDM5C* gene mutation may be due to its relatively high mutation rate in CCRCC compared to the other two X chromosome encoded genes assessed, *KDM6A* and *STAG2*, or due to mutation of this gene having a greater effect on CCRCC tumorigenesis than the other two. Interestingly, a recent publication demonstrated tumor heterogeneity in two CCRCC tumors where one tumor from a male patient had the same *VHL* and *PBRM1* mutations in all regions but had gained different *KDM5C* mutations in different tumor regions demonstrating the repeated importance of losing expression of this gene [8]. Additionally, a recent report suggested that the loss of *KDM5C* activity has a greater tumorigenic effect in cells that have lost *VHL* which may explain the higher rate of *KDM5C* mutation in the frequently *VHL* mutated CCRCCs [9].

A further factor concerning X chromosome encoded genes is the effect of X inactivation in female patients. Although the majority of genes on the X chromosome are subject to inactivation of one allele, both *KDM5C* and *KDM6A* are examples of genes that escape X inactivation, therefore resulting in two active copies in female cells that would need to be biallelically inactivated in a similar manner to any autosomal gene to match a single mutation in a male tumor [10–12]. This is possibly due to the existence of homologues of these two genes on the Y chromosome with *KDM5D* demonstrating an ~85% identity to *KDM5C* and *UTY* demonstrating an ~83% identity to *KDM6A* (*UTX*) gene. These genes may vary in their abilities to compensate for loss of the X chromosome versions and the so the increases in *KDM5C* mutation but not *KDM6A* mutation in males may be due to the inability of *KDM5D* to compensate for *KDM5C* loss. Few studies have assessed this, but in murine neurons it has been shown that *KDM5D* is expressed at lower levels than *KDM5C* [13]. The observation of a gender bias in *KDM5C* mutation was observed in all three cohorts demonstrating no differences due to the differing ethnical background of each cohort, but this observation should be investigated in further cohorts to confirm this correlation.

The observation that mutations of *PBRM1* or *BAP1* are more common in males and females respectively seems less logical as they are both present on chromosome 3p rather than a sex chromosome. Similarly to *KDM5C*, both *PBRM1* and *BAP1* are chromatin remodelers/modifiers and the loss of each gene is thought to have differing effects on mRNA expression profiles, but result in similar overall effects such that a degree of mutual exclusivity is observed between these mutations. Analysis of the CCRCC TCGA cohort demonstrated that loss of these chromatin modifiers produces different mRNA profiles, although loss of multiple genes was observed in some tumors [2]. *PBRM1*, *BAP1* and *KDM5C* represent chromatin modifying or remodeling genes and thus their loss should affect the mRNA expression profiles within the cells. It has been demonstrated that the normal kidney tissue in male and female rats express differing mRNA profiles and proposed that this might contribute to the molecular mechanism than produces the gender-related differences in renal disease incidence in humans [14]. The loss of chromatin remodeling/modifier genes may therefore have differing effects within this different backgrounds and potentially, the loss of a specific gene may be more advantageous to a tumor that is exposed to the basic physiology of one gender in comparison to the other. Thus, even if mutation of any one of these three genes is equally as likely, the selection pressure for mutation of a specific gene will differ between the genders due to the more beneficial effects of the loss of that gene in a specific gender. It is possible that this is the major driving force for the increased *KDM5C* mutation frequency in males and its position on the X chromosome is simply an additional advantage to its increased mutation frequency. The convergence of gender specific mRNA expression and effect of loss of a chromatin modifier gene may also explain the observation that *BAP1* mutations in female patients predict for significantly poorer survival, but not in male patients. This observation could be very important, but needs to be further confirmed in larger cohorts. This observation may also be dependent upon the ethnic background of the assessed cohort as the female bias for *BAP1* mutation was only observed in the TCGA cohort and it was this cohort that provided the survival analysis. Assuming this effect is confirmed in further studies, it may be very important for modifying therapy based on personalized medicine as females with *BAP1* mutations may require more immediate and aggressive therapeutic intervention. Additionally, if targeted therapies are identified for *BAP1* mutated RCCs the gender of the clinical trial participants should be considered when the trial is planned and analyzed.

In summary, gender specific mutation of genes important for tumorigenesis is evident in CCRCC and the combination of gender and mutation of a specific gene, such as *BAP1*, may affect the overall survival of the patient. These findings could affect interpretation of genetic alterations in patients with CCRCC and could provide insight into the role of gender factors in initiation or progression of this and other malignancies.

## Supporting Information

S1 Fig Mutual Exclusivity of Common CCRCC-Associated Mutations. (A) Mutual exclusivity of 10 genes (*KDM5C*, *KDM6A*, *STAG2*, *VHL*, *PBRM1*, *BAP1*, *SETD2*, *ARID1A*, *PTEN* and *TP53*) commonly mutated in the CCRCC TCGA cohort was analyzed using the the cBioPortal software (http://www.cbioportal.org/index.do) [6,7]. (B) This data was separated by gender to produce oncoprints with green boxes designating mutation.
(TIF)

S2 Fig Gender and Mutation Specific Survival Analysis. (A) Kaplan-Meier survival analysis graphs for the CCRCC TCGA cohort were produced comparing samples based on Gender, (B) with or without mutation of *PBRM1* and (C) comparing the differences in survival with or within mutation of *PBRM1* or *KDM5C* for each gender. Analysis was performed using the cBioPortal software (http://www.cbioportal.org/index.do) [6,7] and mutated samples were represented by red lines and wild-type samples by blue lines. p-values less than 0.01 were considered statistically significant.
(TIF)

S1 Table Mutations identified in the TCGA KIRC, Sato *et al*, and Guo *et al* cohorts for 10 Genes Commonly Mutated in Clear cell Renal Cell Carcinoma. (XLSX)

S2 Table Analysis of Mutation Frequency in 8 Genes Commonly Mutated in Bladder Cancer. (XLSX)
