# Supplementary figures and images for: Gender Specific Mutation Incidence and Survival Associations in Clear Cell Renal Cell Carcinoma (CCRCC)
Source: PLoS One. 2015 Oct 20;10(10):e0140257. doi: 10.1371/journal.pone.0140257 (PMC4618848; doi:10.1371/journal.pone.0140257)

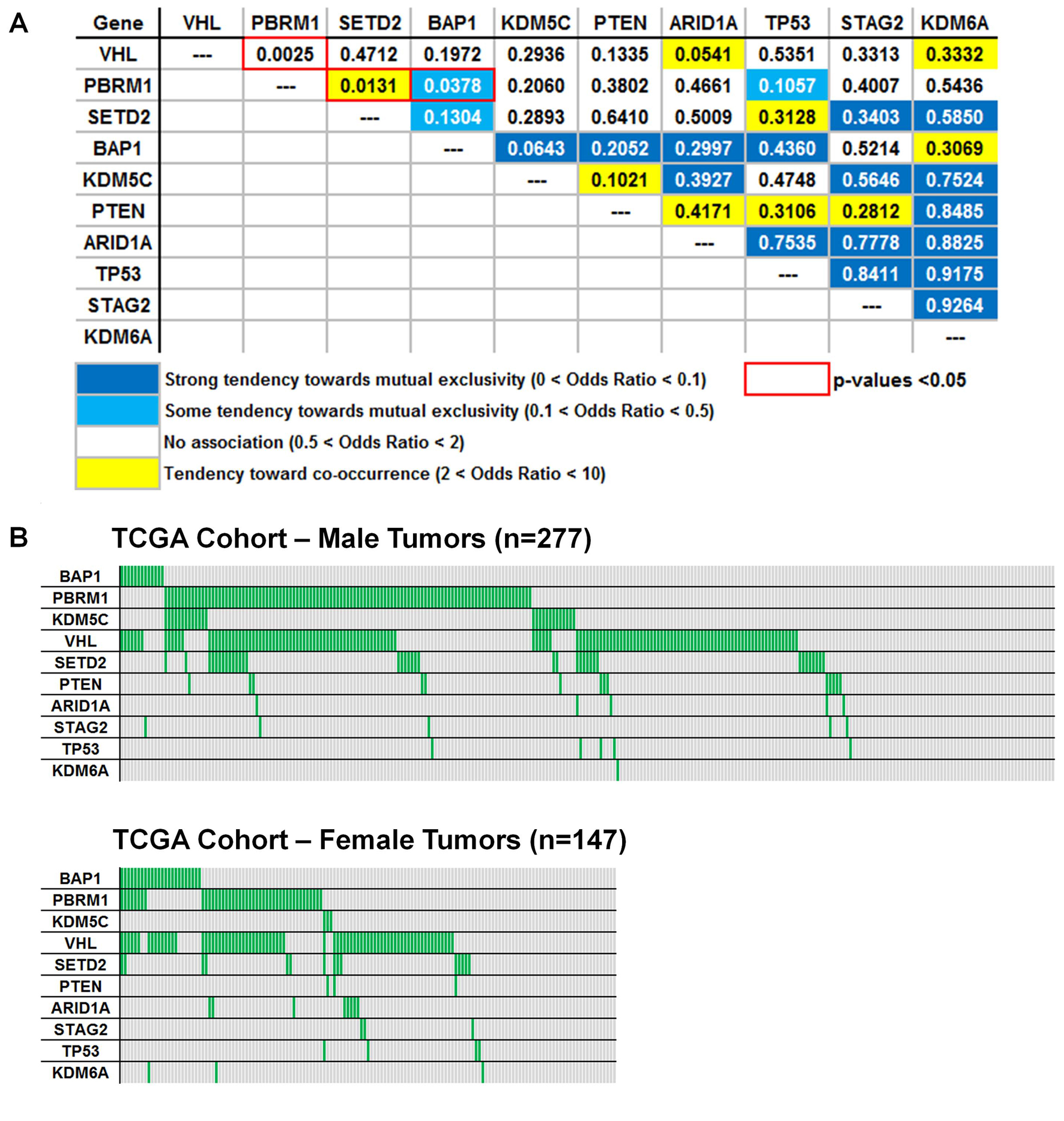

Supplement: S1 Fig — (A) Mutual exclusivity of 10 genes (KDM5C, KDM6A, STAG2, VHL, PBRM1, BAP1, SETD2, ARID1A, PTEN and TP53) commonly mutated in the CCRCC TCGA cohort was analyzed using the the cBioPortal software (http://www.cbioportal.org/index.do) [6,7]. (B) This data was separated by gender to produce oncoprints with green boxes designating mutation. (TIF) [file pone.0140257.s001.tif]

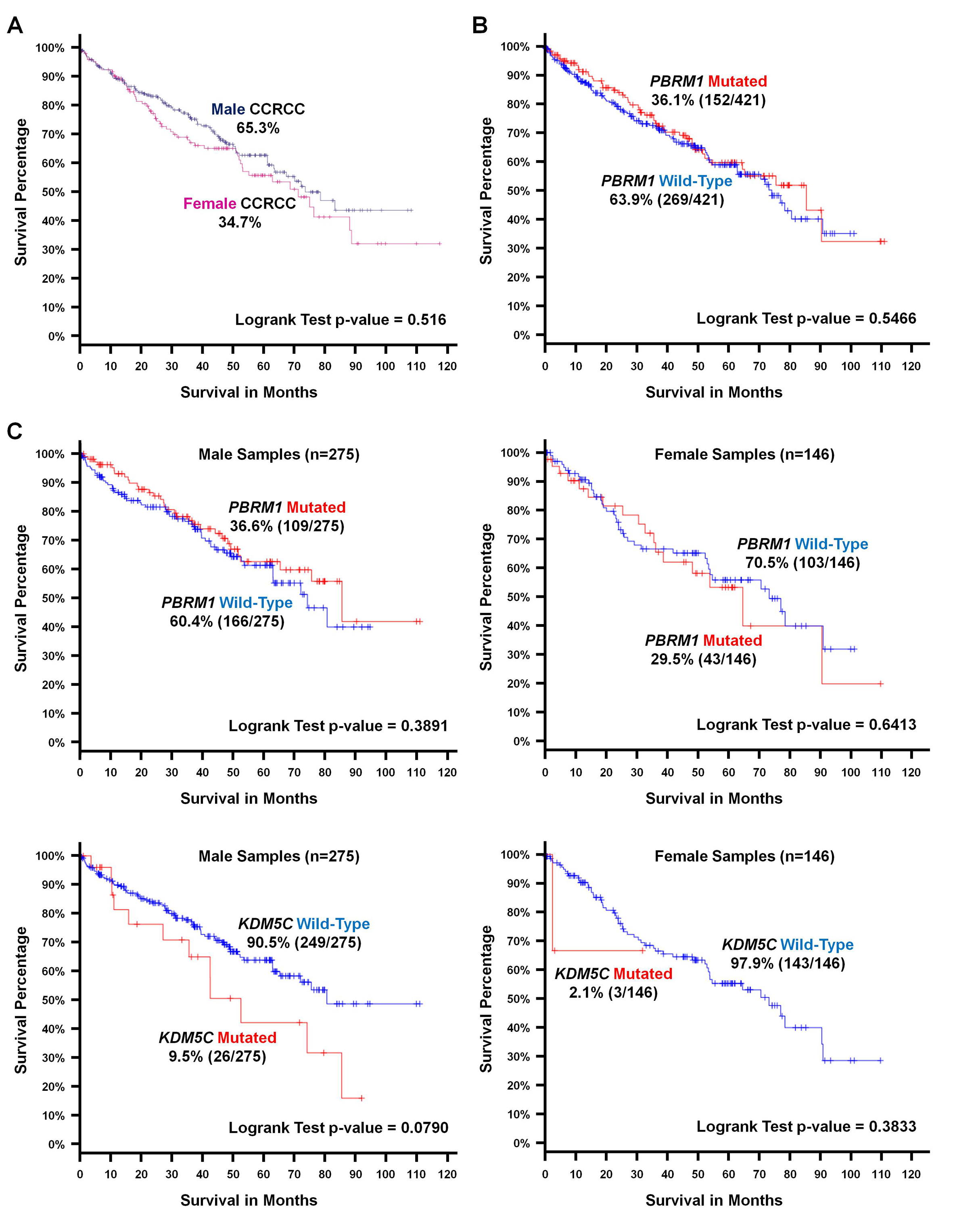

Supplement: S2 Fig — (A) Kaplan-Meier survival analysis graphs for the CCRCC TCGA cohort were produced comparing samples based on Gender, (B) with or without mutation of PBRM1 and (C) comparing the differences in survival with or within mutation of PBRM1 or KDM5C for each gender. Analysis was performed using the cBioPortal software (http://www.cbioportal.org/index.do) [6,7] and mutated samples were represented by red lines and wild-type samples by blue lines. p-values less than 0.01 were considered statistically significant. (TIF) [file pone.0140257.s002.tif]
